# Synthesis and Characterization of Transition Metal Complexes Supported by Phosphorus Ligands Obtained Using Hydrophosphination of Cyclic Internal Alkenes

**DOI:** 10.3390/molecules29163946

**Published:** 2024-08-21

**Authors:** Victoria Mechrouk, Damien Bissessar, Julien Egly, Jordan Parmentier, Stéphane Bellemin-Laponnaz

**Affiliations:** Institut de Physique et Chimie des Matériaux de Strasbourg, Université de Strasbourg-CNRS UMR7504, 23 rue du Loess, BP 43, CEDEX 2, 67034 Strasbourg, France

**Keywords:** phosphorus ligand, hydrophosphination, coordination chemistry, homogeneous catalysis

## Abstract

The design and study of rich, bulky phosphorus ligands is a key area of research for homogeneous catalysis. Here, we describe an original strategy using a hydrophosphination reaction to produce phosphines of interest for coordination chemistry and homogenous catalysis. In particular, the phosphine obtained by reacting diphenylphosphine with acenaphthylene (ligand **2**) gives a ligand that adopts an unusual spatial geometry. The coordination chemistry of the ligand has been investigated with Au(I), Ag(I), Cu(I), and Pd(II), for which a complete characterization could be made, particularly in X-ray diffraction studies. The reactivity of some of these complexes has been demonstrated, particularly in Pd-catalyzed cross-coupling reactions and Au-catalyzed hydroaminations and in the hydration of alkynes.

## 1. Introduction

**Figure 1:**
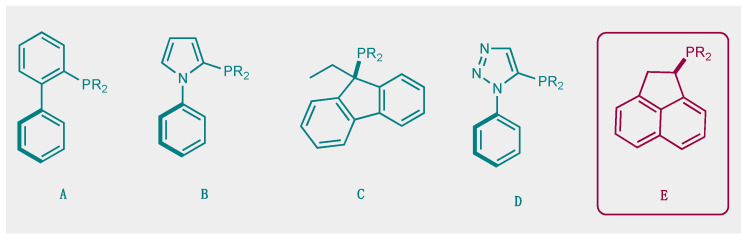
Selected examples of bulky electron-rich phosphines (**A**–**D**) and molecular structure of (**E**).

The development of efficient metal-catalyzed reactions has benefited from the ongoing development of new bulky phosphines, particularly in cross-coupling reactions [1]. As early as the mid-1980s, Osborn highlighted the impact that phosphine steric hindrance could have on the reactivity of palladium to catalytically activate chlorobenzene, for example [2]. Buchwald’s major contributions from the late 1990s onwards developed the synthetic chemistry offered by new, original, and bulky ligands **A**, Figure 1 [3]. These bulky, electron-rich phosphine ligands, which can significantly improve the efficiency and selectivity of cross-coupling reactions, are characterized by structural features brought about by the combination of alkyl groups and a biaryl moiety on the phosphorus. The ligands developed by this group are an often necessary part of the toolbox of today’s synthetic chemists. Since the first Buchwald examples and all of the improved versions of this family of ligands, it appears that this type of molecular architecture, combining a biaryl group in which the lower ring is positioned perpendicular to the first ring, offers many advantages for catalysis. For example, it allows for an ML species to be favored over a homoleptic ML_2_ species, and the second ring also allows for stabilizing Pd-arene interactions. In addition, these ligands have been widely shown to be of interest with other metals, notably gold(I) catalysis, giving rise to transformations otherwise unattainable under the action of other gold(I) catalysts [4].

This type of molecular structure, with an aromatic ring positioned perpendicular to the first ring and thus close to the phosphorus, is a source of inspiration for the design of new ligands. By way of example, we can cite the work of Beller and co-workers who developed dialkyl−2-(N-arylindolyl)phosphines **B** [5], the work of Plenio and Fleckenstein [6,7] based on 9-fluorenyldialkylphosphines derivatives **C**, and, more recently, of Fossey et al., who developed a series of 1-phenyl−5-phosphino 1,2,3-triazoles **D** [8]. 

Here, we wish to describe the synthesis and study of the coordination chemistry of an original and novel phosphine with structural features that could be of interest for the development of homogeneous phosphine-based catalysts **E**. This type of molecular structure is very easily accessible using the direct hydrophosphination of alkenes and can be carried out in very large quantities. We also describe first coordination chemistry results with Pd(II), Au(I), and Ag(I), as well as some results in homogeneous catalysis.

## 2. Results

### 2.1. Phosphine Synthesis

**Figure 2:**
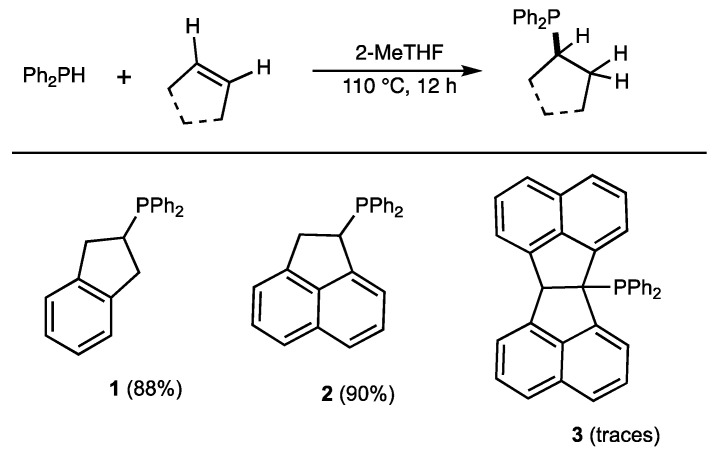
Catalyst-free hydrophosphination of alkenes. Reaction condition: alkene (1 equiv.), diphenylphosphine (1.1 equiv.), 2-MeTHF (4 equiv.) under argon.

The phosphine ligands **1** and **2** were synthesized using direct hydrophosphination of the corresponding alkene [9]. The following reaction condition was used: alkene (1 equiv.), Ph_2_PH (1.1 equiv.), and 2-Methyl THF (4 equiv.) under argon in a closed vessel at 110 °C for 12 h (Figure 2). The product was purified using flash column chromatography under an inert atmosphere, with yields of around 75% in both cases. Alternatively, direct precipitation by addition of *n*-hexane under nitrogen gave better results: the phosphine **1** was isolated in 88% yield and **2** in 90% yield. Attempts to generate phosphine **3** were unsuccessful, and only traces of the expected product were observed in the reaction mixture. 

The two phosphines were characterized using classical techniques and were found to be sensitive when exposed to air. For example, phosphine **2**, in its solid state, was completely oxidized to the corresponding phosphine oxide after 11 days in air. Under the same conditions, 40% of phosphine **1** was converted to phosphine oxide. We recrystallized the two compounds to obtain single crystals for X-ray diffraction studies. The molecular structure of the phosphine oxide of **1** and **2** could be established in the solid state (see Supplementary Materials for details).

### 2.2. Synthesis and Characterization of Transition Metal Complexes

#### 2.2.1. Synthesis

By reacting the metal precursor with a stochiometric amount of phosphine (**1** or **2**), the corresponding transition metal complex was formed. In all cases, the reaction was performed in dry dichloromethane or dry diethylether at room temperature to 50 °C under argon atmosphere for 0.5 h to 12 h. All products were isolated as air stable solids and were characterized using classical analytical methods. The ^31^P{^1^H} NMR spectra showed a signal slightly shifted downwards with respect to the free ligand, confirming the formation of the metal complexes.

#### 2.2.2. Crystal Structure of the Metal Complexes

##### Complex **1_Au_**

**Figure 3:**
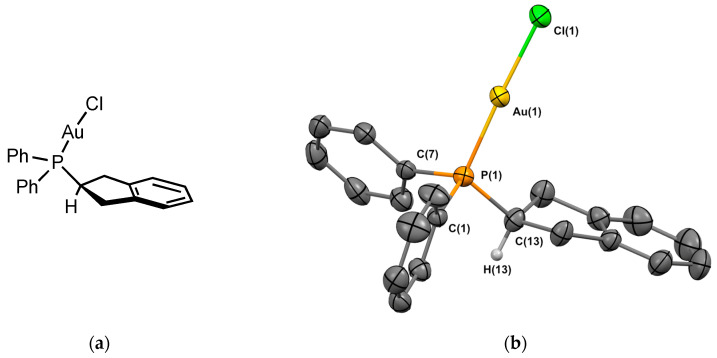
(**a**) Molecular structure of **1_Au_**; (**b**) single-crystal X-ray molecular structure of gold complex **1_Au_**. Hydrogen atoms are omitted for clarity, with the exception of the sp^3^ carbon next to the phosphorus. Selected bond distances (Å) and angles (deg): P(1)-Au(1), 2.2333(10); P(1)-C(1), 1.814(4), P(1)-C(7), 1.813(4), P(1)-C(13), 1.826(4), Au(1)-Cl(1), 2.2914(12), C(1)-P(1)-C(7), 104.29(18), C(1)-P(1)-C(13), 105.40(18) C(7)-P(1)-C(13), 107.74(19), Au(1)-P(1)-C(1), 113.36(13), Au(1)-P(1)-C(7), 114.24(13), Au(1)-P(1)-C(13), 111.17(15), P(1)-Au(1)-Cl(1), 177.41(4).

Gold chloride complex **1_Au_** was prepared using a reaction of the ligand with [(Me_2_S)AuCl] in 82% yield. Crystals of **1_Au_** suitable for X-ray crystallography were obtained from CH_2_Cl_2_/*n*-hexane. The molecular structure of the complex is depicted in Figure 3. Complex **1_Au_** was crystallized in the monoclinic P 2_1_/c space group. The P-Au bond length is 2.233 Å, which is in the range of the analogous Johnphos gold(I) complexes [10,11]. The P–Au–Cl angle is 177.6°, similar to those observed in analogous phosphine complexes. The hydrogen of the sp^3^ carbon next to the phosphorus is oriented away from the metal. The two hydrogens of the cyclopentene pointing towards the metal center are located at a distance of 3.1 Å, suggesting a weak intramolecular CH⋯Au interaction, although the distance is above than the sum of their Van der Waals radii (2.86 Å).

##### Complex **2_Au_**

**Figure 4:**
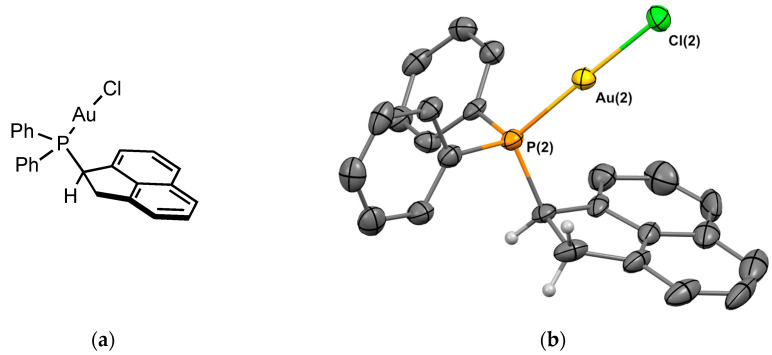
(**a**) Molecular structure of **2_Au_**; (**b**) single-crystal X-ray molecular structure of gold complex **2_Au_**. Hydrogen atoms are omitted for clarity, with the exception of the sp3 carbons next to the phosphorus. Selected bond distances (Å) and angles (deg): P(1)-Au(1), 2.239(2); P(1)-C(1), 1.814(9), P(1)-C(7), 1.824(9), P(1)-C(13), 1.838(8), Au(1)-Cl(1), 2.300(2), C(1)-P(1)-C(7), 104.0(4), C(1)-P(1)-C(13), 105.7(4), C(7)-P(1)-C(13), 105.7(4), Au(1)-P(1)-C(1), 114.8(3), Au(1)-P(1)-C(7), 113.2(3), Au(1)-P(1)-C(13), 112.5(3), P(1)-Au(1)-Cl(1), 177.74(8).

Gold chloride complex **2_Au_** was prepared using a reaction of the ligand with [(Me_2_S)AuCl] in 78% yield. Crystals of **2_Au_** suitable for X-ray crystallography were obtained from CH_2_Cl_2_/*n*-hexane. The molecular structure of the complex is depicted in Figure 4. The complex was crystallized in the monoclinic P 2_1_/c space group, and the asymmetric unit contains the two enantiomers. Figure 4 shows one of the two enantiomers present in the asymmetric unit. The P-Au bond length is 2.239 Å, and as in the previous structure, the C-H of the sp^3^ carbon is oriented in the opposite direction to the metal. The P–Au–Cl angle is 178.5°.

##### Complex **2_Ag_**

**Figure 5:**
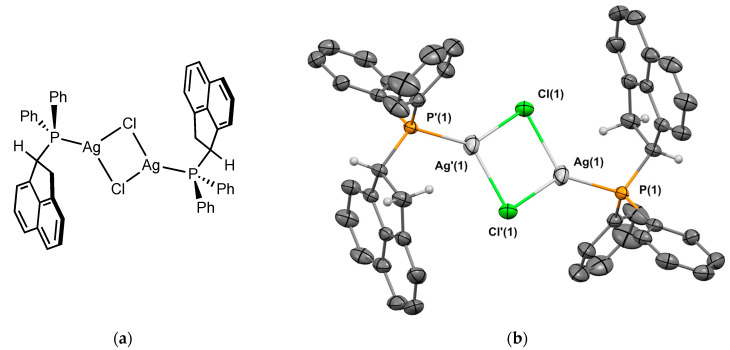
(**a**) Molecular structure of **1_Ag_**; (**b**) single-crystal X-ray molecular structure of silver complex **1_Ag_**. Hydrogen atoms are omitted for clarity, with the exception of the sp^3^ carbons next to the phosphorus. Selected bond distances (Å) and angles (deg): P(1)-Ag(1), 2.3580(6), P(1)-C(1), 1.823(2), P(1)-C(7), 1.820(2), P(1)-C(13), 1.840(2), Ag(1)-Cl(1), 2.5055(5), Ag(1)-Cl’(1), 2.5582(6) Ag(1)-Ag’(1), 3.3444(4), Cl(1)-Ag(1)-P(1), 135.73(2) Cl’(1)-Ag(1)-P(1), 126.43(2), Ag(1)-Cl(1)-Ag(1), 82.665(18), Ag(1)-P(1)-C(1), 112.65(7), Ag(1)-P(1)-C(7), 115.85(7), Ag(1)-P(1)-C(13), 113.63(7), C(1)-P(1)-C(7), 103.78(10), C(1)-P(1)-C(13), 105.37(10), C(7)-P(1)-C(13), 104.47(10).

Silver chloride complex **2_Ag_** was prepared using a reaction of the **2** with an excess of silver chloride (50 °C for 48 h) in 44% yield. Crystals of **2_Ag_** suitable for X-ray crystallography were obtained from CH_2_Cl_2_/*n*-hexane. The molecular structure of the complex is depicted in Figure 5. Complex **2_Ag_** was crystallized in the triclinic P1 space group. The molecule is a dimer [{**2**–Ag(μ-Cl)}_2_] with Ag-Cl-Ag bridges, and is in the heterochiral form. The P-Ag bond length is 2.358 Å, which is in the range of the analogous Johnphos silver(I) complexes [12]. The Ag⋯Ag distance is 3.344 Å. Regarding the conformation of the ligand, the C-H of the sp^3^ carbon next to the phosphorus is again oriented in the opposite direction to the metal.

##### Complex **2_Cu_**

**Figure 6:**
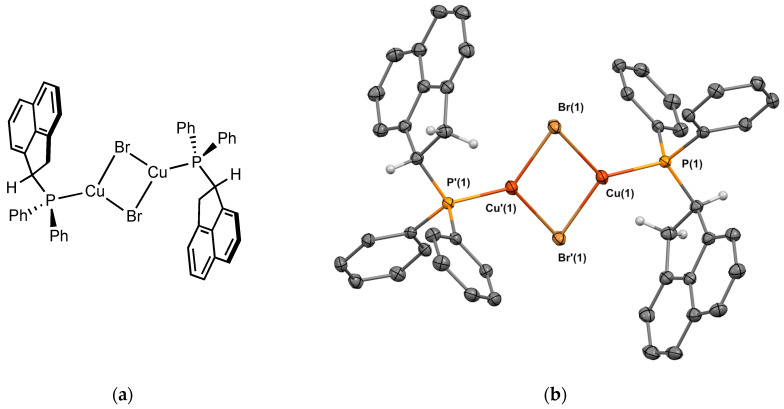
(**a**) Molecular structure of **2_Cu_**; (**b**) Single-crystal X-ray molecular structure of copper complex **2_Cu_**. Hydrogen atoms are omitted for clarity, with the exception of the sp^3^ carbons next to the phosphorus. Selected bond distances (Å) and angles (deg): P(1)-Cu(1), 2.1952(4), P(1)-C(1), 1.8253(14), P(1)-C(7), 1.8230(13), P(1)-C(13), 1.8398(13), Cu(1)-Br(1), 2.4228(2), Cu(1)-Br’(1), 4.4251 (2), Cu(1)-Cu’(1), 2.8801(3), Br(1)-Cu(1)-P(1), 124.526(12), Br’(1)-Cu(1)-P(1), 125.347(12) Cu(1)-Br(1)-Cu(1), 72.894(7), Cu(1)-P(1)-C(1), 108.74(4), Cu(1)-P(1)-C(7), 118.39(4), Cu(1)-P(1)-C(13), 116.73(4), C(1)-P(1)-C(7), 103.11(), C(1)-P(1)-C(13), 103.76(6), C(7)-P(1)-C(13), 104.37(6).

The copper bromide complex **2_Cu_** was obtained using [(Me_2_S)CuBr] and was isolated in 42% yield as a white powder. Crystals suitable for X-ray crystallography were obtained from CH_2_Cl_2_/*n*-pentane (Figure 6). The structure is isostructural to the structure obtained with silver **1_Ag_**. The molecule is a dimer with Cu-Br-Cu bridges, and the ligand is in the heterochiral form. The P-Cu bond length is 2.195 Å. The Cu⋯Cu distance is 2.880 Å, almost equal to the sum of the copper van der Waals radii (2.8 Å), indicating minimal interaction between the two copper ions.

##### Complex **2_Pd1_**

**Figure 7:**
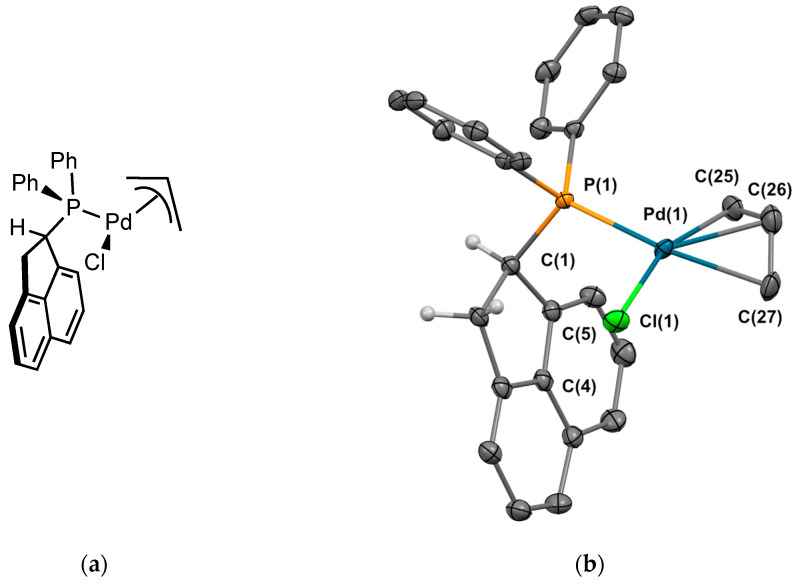
(**a**) Molecular structure of **2_Pd1_**; (**b**) single-crystal X-ray molecular structure of palladium complex **2_Pd1_**. Hydrogen atoms are omitted for clarity, with the exception of the sp^3^ carbons next to the phosphorus. Selected bond distances (Å) and angles (deg): P(1)-Pd(1), 2.2988(7), P(1)-C(1), 1.841(3), P(1)-C(13), 1.830(3), P(1)-C(19), 1.830(3), Pd(1)-Cl(1), 2.3658(7), Pd(1)-C(25), 2.118(3), Pd(1)-C(26), 2.201(3), Pd(1)-C(27), 2.201(3), Cl(1)-Pd(1)-P(1), 99.87(2), Pd(1)-P(1)-C(1), 110.94(9), Pd(1)-P(1)-C(13), 117.23(9), Pd(1)-P(1)-C(19), 117.42(9), C(1)-P(1)-C(13), 103.26(12), C(1)-P(1)-C(19), 105.59(12), C(13)-P(1),C(19), 100.78(12).

Reaction of [Pd(allyl)Cl]_2_ with two equivalents of phosphine **2** in diethylether overnight gave the corresponding complex **2_Pd1_** in 86% yield [13]. Crystals suitable for X-ray crystallography were obtained from CH_2_Cl_2_/*n*-pentane (Figure 7). The complex was crystallized in the monoclinic P 2_1_/c space group. The palladium atom adopts a planar coordination geometry with the Π-allyl moiety, the halogen, and the phosphorus. The Pd-P and Pd-C bond lengths are within the range found for related complexes reported in the literature [14]. The P-Pd bond length is 2.299 Å, and as in the previous structure, the C-H of the sp^3^ carbon is oriented in the opposite direction to the palladium center. The orientation of the allyl group is endo, endo being defined as the central C-H allylic bond pointing in the other direction as the 1,2-dihydroacenaphthylene moiety. We noted the presence in a solution of a minor isomer, probably the allyl group oriented in the other direction, as deduced from the NMR studies.

#### Complex **2_Pd2_**

**Figure 8:**
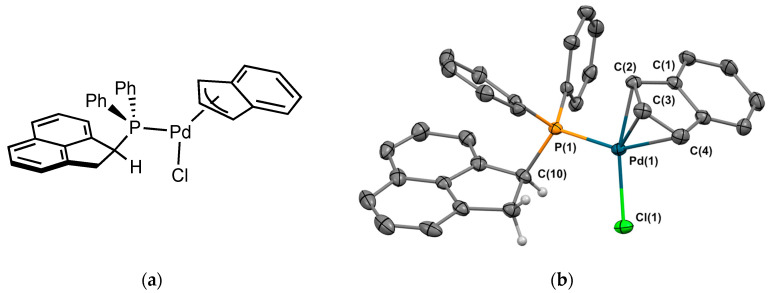
(**a**) Molecular structure of **2_Pd2_**; (**b**) single-crystal X-ray molecular structure of palladium complex **2_Pd1_**. Hydrogen atoms are omitted for clarity, with the exception of the sp3 carbons next to the phosphorus. Selected bond distances (Å) and angles (deg): P(1)-Pd(1), 2.2622(6), P(1)-C(10), 1.847(2), P(1)-C(22), 1.820(2), P(1)-C(28), 1.818(2), Pd(1)-Cl(1), 2.3558(6), Pd(1)-C(1), 2.581(2), Pd(1)-C(2), 2.132(2), Pd(1)-C(3), 2.216(2), Pd(1)-C(4), 2.376(3), Cl(1)-Pd(1)-P(1), 106.41(6), Pd(1)-P(1)-C(10), 113.89(8), Pd(1)-P(1)-C(22), 112.47(8), Pd(1)-P(1)-C(28), 112.36(8), C(10)-P(1)-C(22), 107.76(11), C(10)-P(1)-C(28), 103.56(11), C(22)-P(1)-C(28), 106.12(11).

The palladium complex **2_Pd2_** with an indenyl moiety instead an allyl moiety was prepared from the precursor [Pd(indenyl)Cl]_2_, prepared according to Hazari and coll. [15]. The expected product was isolated in 68% yield as a brown-orange solid. Figure 8 displays the molecular structure of the compound. The coordination around the metal is a square planar. An interesting structural feature is that the indenyl fragment binds to the metal center with a hapticity intermediate between η^3^ and η^5^. The five Pd-C bond lengths range from 2.13 to 2.68 Å. Another important feature is the orientation of the 1,2-dihydroacenaphthylene fraction, which points away from the metal center. As a consequence, for the first time, the C-H of the sp^3^ carbon next to the phosphorus is oriented in the same direction as the metal.

### 2.3. Preliminary Results in Catalysis

#### 2.3.1. Palladium Catalysis

We tested the activity of ligand **2** in the Suzuki–Miyaura cross-coupling reaction in order to assess its catalytic potential (Table 1, Entries 1–3). Standardized reaction conditions regarding solvent and choice of base were applied (1 mol% of **2**, 0.5 mol% [Pd(allyl)Cl]_2_, 4 equiv. of K_2_CO_3_, THF/water 8/2, 80 °C, 12 h).

The coupling of phenyl boronic acid Ph-B(OH)_2_ with *p*-nitrochlorobenzene, *p*-chloroacetophenone, or *p*-methoxychlorobenzene gave the corresponding products in 96%, 99%, and 98%, respectively. Lowering the catalyst loading gave poor results in terms of yields. In the Buchwald–Hartwig coupling reaction, under standard conditions (i.e., 2 mol% of **2**, 1 mol% Pd(OAc)_2_, NaO*^t^*Bu 1.2 equiv. in toluene at 110 °C for 18 h), only bromoaryl derivatives were activated [16]. For example, the reaction of diphenylamine with bromobenzene gave the triaryl amine product in 30% yield (Entry 4). Using the *p*-nitrobromobenzene, the corresponding product was isolated in 44% yield (Entry 5).

#### 2.3.2. Gold Catalysis

Gold(I) complexes associated with Buchwald-type ligands have shown great potential, and these systems can provide unrivaled catalytic performance in a number of chemical reactions of interest [17]. Gold-catalyzed intermolecular hydroamination of alkynes is now a well-established reaction, and we studied the reactivity of the gold complex **2_Au_** after activation using chloride abstraction [18]. The complex **2_Au_** was first activated to generate the corresponding phosphine gold(I) bis-(trifluoromethanesulfonyl)imidate complex (**2**)AuNTf_2_, which was fully characterized (see Supplementary Materials) [19]. The complex was active in the hydroamination of phenylacetylene, as shown in Equations (1) and (2). With primary amine PhNH_2_ (Equation (1)), 94% of the corresponding product was obtained under our experimental conditions. With secondary amine, the corresponding product was isolated in 39% yield.

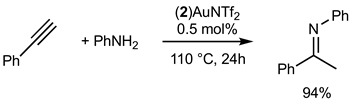
(1)

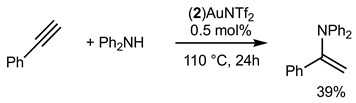
(2)

The hydrofunctionalisation reaction of alkynes with water can be catalyzed with gold and generally give Markovnikov regioselectivity in the case of terminal alkynes. Bulky phosphine ligands are known to efficiently catalyze such reactions. Equation (3) displays the catalytic hydration of phenylacetylene with 5 mol% of catalyst. Only 5% of the ketone product was isolated. Interestingly, we found that with the addition of a donating secondary amine such as Ph2NH, the efficiency of the reaction was increased up to 47%.

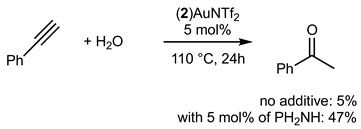
(3)

## 3. Discussion

The hydrophosphination reaction is a very simple method for generating tertiary phosphines in one step, and generally in very good yields [9,20,21]. If the reaction is carried out on a cyclic internal alkene, then the reaction product could have an interesting molecular structure suitable for the development of phosphine ligands for transition metal catalysis. This leads to the formation of a cyclic moiety that could be positioned close to the phosphorus atom to form monoligated complexes of ML. For example, the hydrophosphination reaction of indene leads to the selective formation of phosphine **1** in 88% yield (Figure 2), which contains a 2,3-dihydro−1H-indene group in the 2-position [22]. When this reaction is carried out with acenaphthylene as the substrate, the corresponding phosphine **2** contains a 1,2-dihydroacenaphthylene group and is therefore a chiral racemic product (Figure 2, 90%). Acenaphtho[1,2-a]acenaphthylene was also tested as a substrate, but proved unreactive under our experimental conditions.

We anticipated that this type of molecular structure could be interesting as a ligand for transition metals, particularly in view of what has been described with Buchwald-type ligands. We therefore set out to study the coordination chemistry of these ligands, especially ligand **2**, in order to investigate the molecular structure of the resulting complexes. Complexes with gold(I), silver(I), copper(I), and palladium(II) have been synthesized and characterized, including X-ray diffraction studies. A general feature of all of these molecular structures is the positioning of the 1,2-dihydroacenaphthylene in relation to the metal, except for the Pd complex bearing bulky indenyl ligand **2_Pd2_**. In these complexes, the dihydroacenaphthylene appears close to the first coordination sphere.

**Figure 9:**
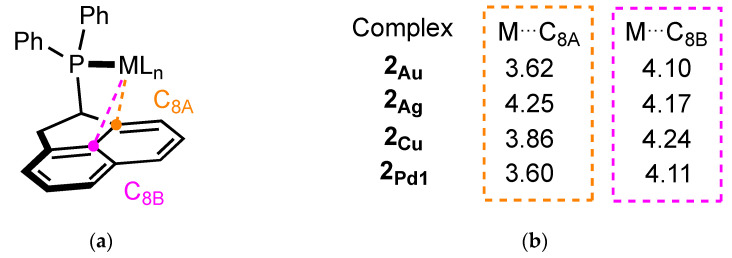
(**a**) Description of M-C distances; (**b**) average carbon–metal distances measured from the X-ray structures.

In comparison, Buchwald-type ligands are known to promote the formation of highly reactive monoligated complexes by positioning the second ring in the first coordination sphere of the metal, preventing the attachment of multiple ancillary ligands. In addition, they can also stabilize the unsaturated center of the metal through interactions with the π-system of the phosphine (pseudobidentate binding mode) [23,24]. Figure 9 displays the average carbon–metal distances measured from the X-ray structures. They range from 3.60 Å to 4.25 Å for the C_8A_ atom and from 4.10 to 4.24 Å for the central carbon C_8B_, which is considerably longer than the distances measured in the Johnphos-type complexes (from 2.35 Å with Pd to 3.16 Å with Au, for the C_ipso_-M bond length) [12,13,25,26]. However, one notable difference between JohnPhos and ligand 2 is the presence of very bulky substituents (*^t^*Bu vs. Ph) on the phosphorus, which can have a significant impact on the properties of the ligand. Replacing the two Ph substituents with more sterically demanding groups, such as *^t^*Bu or Cy on ligand (**2**), can have an impact on the geometry and properties of the final complex.

Finally, we studied the reactivity of catalytic systems based on ligand **2**. Interesting results were obtained in the Pd-catalyzed cross-coupling reactions, but these did not reach the best systems for this type of reaction. Ligand **1** gave very poor results in comparison. The gold complex has also been tested in hydroamination and hydration reactions with mixed results. A notable finding was obtained in the alkyne hydration reaction, where a catalytic amount of secondary amine was found to greatly improve reaction yield (from 5% to 47%). However, these results are still modest compared with other phosphine-based systems [27,28].

## 4. Materials and Methods

### 4.1. General Remarks

All of the synthetic steps of the air- and moisture-sensitive compounds were carried out using standard Schlenk techniques under an argon atmosphere, and the solvents were purified and degassed following standard procedures. Crystallization of **1** and **2** for X-ray diffraction analysis was carried out in air. All of the reagents were purchased from commercial chemical suppliers and were used without further purification. ^1^H and ^13^C nuclear magnetic resonance (NMR) spectra were recorded on a Bruker AVANCE 300 or Bruker AVANCE 500 spectrometer using the residual solvent peak as a reference (CDCl_3_: δ_H_ = 7.26 ppm; δ_C_ = 77.16 ppm) at 295 K. Positive mode electrospray ionization mass spectra (ESI-HRMS) analyses were carried out on a microTOF, Bruker Daltonics, Billerica M.A., USA.

### 4.2. X-ray Analyses

The crystals were placed in oil, and a single crystal was selected, mounted on a glass fiber, and placed in a low-temperature N_2_ stream. X-ray diffraction data collection was carried out on a Bruker PHOTON III DUO CPAD diffractometer equipped with an Oxford Cryosystem liquid N_2_ device, using Mo-Kα radiation (λ = 0.71073 Å) for compounds **2_Cu_**, **2_Pd1_**, and **2_Pd2_**. For complex **2_Au_**, X-ray diffraction data collection was carried out on a Nonius Kappa-CCD diffractometer equipped with an Oxford Cryosystem liquid N_2_ device using Mo-Kα radiation (λ = 0.71073 Å). For complexes **1_Au_**, **2_Ag_**, and ligands, X-ray diffraction data collection was carried out on a Bruker APEX II DUO Kappa-CCD diffractometer (Brucker, Wissembourg, France) equipped with an Oxford Cryosystem liquid N_2_ device using Mo-Kα radiation (λ = 0.71073 Å). CCDC deposit numbers: 2373136−2373143.

### 4.3. Synthesis of Ligand **1**

A mixture of indene (1.0 g; 8.61 mmol), diphenylphosphine (1.92 g; 10.3 mmol), and 2Me-THF (4.0 mL) was stirred at 110 °C under an argon atmosphere for 12 h. The crude product was purified using the addition of *n*-hexane Phosphine **1** (2.28 g: 88%) was obtained as a white powder. Crystals of phosphine oxide suitable for X-ray crystallography were obtained from slow CH_2_Cl_2_ evaporation in open air.

^1^H NMR (500 MHz, CDCl_3_): δ = 2.88–3.15 (m, 4H); 3.24 (tt, J = 8.0 Hz, 8.0 Hz, 1H); 7.09–7.20 (m, 4H); 7.30–7.41 (m, 6H); 7.48–7.58 (m, 4H) ppm. ^13^C NMR (125 MHz, CDCl_3_): δ = 35.1 (d, J = 39.0 Hz, CH_2_); 39.2 (d, J = 7.3 Hz, CH); 127.1 (s, CH); 128.8 (s, CH); 129.2 (s, C); 130.5 (d, J = 11.2 Hz, CH); 133.7 (s, CH); 134.4 (d, J = 12.7 Hz, CH); 141.2 (d, J = 9.7 Hz, C) ppm. ^31^P-{^1^H} NMR (202 MHz, CDCl_3_): δ = −5.0 ppm. HRMS (ESI+, *m/z*) [M + H]^+^ calculated 303.1303 found 303.1301.

### 4.4. Synthesis of Ligand **2**

A mixture of acenaphthylene (1.5 g; 9.90 mmol), diphenylphosphine (2.2 g; 11.8 mmol), and 2Me-THF (4.5 mL) was stirred at 110 °C under an argon atmosphere for 12 h. The solvent was removed under reduced pressure and the solid was washed with hexane (trituration under ultrasound). The supernatant was removed and the solid dried under reduced pressure. Phosphine **2** (2.97 g, 90%) was obtained as a white powder.

^1^H NMR (500 MHz, CDCl_3_): δ = 3.19–3.28 (m, 1H); 3.55–3.60 (m, 1H); 4.37–4.42 (m, 1H); 7.22–7.51 (m, 16H) ppm. ^13^C NMR (75 MHz, CDCl_3_) δ 36.45 (d, *J* = 16.2 Hz), 40.55 (d, *J* = 15.1 Hz), 119.35, 120.16 (d, *J* = 5.9 Hz), 122.59, 123.02 (d, *J* = 2.6 Hz), 127.67, 127.89, 128.45 (d, *J* = 1.6 Hz), 128.52, 128.55, 128.78, 129.50, 131.75, 133.05 (d, *J* = 18.2 Hz), 134.56 (d, *J* = 19.8 Hz), 137.22 (d, *J* = 15.4 Hz), 137.67 (d, *J* = 17.3 Hz), 139.45, 143.86, 145.55 (d, *J* = 9.8 Hz) ppm.^31^P NMR (121 MHz, CD_2_Cl_2_) δ −3.3 ppm. HRMS (ESI+, *m/z*) [M + H]^+^ calculated 339.1297 found 339.1290.

### 4.5. Synthesis of Gold Complexes **1_Au_** and **2_Au_**

A solution of the corresponding phosphine (**1** or **2**, 0.59 mmol) and (dimethyl)gold(I) chloride (174 mg; 0.59 mmol) in anhydrous dichloromethane was stirred at room temperature under an argon atmosphere for 30 min. The solvent was evaporated and the solid was washed with hexane (ultrasonic trituration). The supernatant was removed and the solid was dried under reduced pressure. The resulting solid was recrystallized from dichloromethane/*n*-hexane to give the product as a white solid.

**1_Au_**. (290 mg; 82%) ^1^H NMR (500 MHz, CDCl_3_): δ = 3.10–3.17 (m, 2H); 3.23–3.31 (m, 2H); 3.54–3.63 (m, 1H); 7.14–7.18 (m, 4H); 7.48–7.56 (m, 6H); 7.76–7.80 ppm (m, 4H). ^13^C NMR (125 MHz, CDCl_3_): δ = 36.1 (d, J = 41.5 Hz, CH_2_); 37.2 (d, J = 7.3 Hz, CH); 124.0 (s, CH); 126.7 (s, CH); 129.0 (s, C); 129.4 (d, J = 11.5 Hz, CH); 132.1 (s, CH); 134.2 (d, J = 13.0 Hz, CH); 140.8 (d, J = 9.9 Hz, C) ppm. ^31^P-{^1^H} NMR (202 MHz, CDCl_3_): δ = 42.0 (s) ppm. HRMS (ESI+, *m/z*) [M + Li]^+^ calculated 541.0734 found 541.0738.

**2_Au_**. (265 mg; 78%) ^1^H NMR (500 MHz, CDCl_3_): δ = 3.50–3.62 (m, 1H); 3.71–3.82 (m, 1H); 4.86–4.93 (m, 1H); 7.35–7.78 (m, 16H) ppm. ^13^C NMR (125 MHz, CDCl_3_): δ = 36.1 (d, J = 5.3 Hz, CH_2_); 41.4 (d, J = 35.4 Hz; CH); 120.0 (s, CH); 123.2 (s, CH); 125.2 (s, CH); 127.9 (s, CH); 128.1 (s, CH); 129.2 (t, J = 10.9 Hz, CH); 132.0 (s, C); 132.2 (s, CH); 133.2 (s, CH); 133.1 (d, J = 12.5 Hz, CH); 135.1 (d, J = 13.2 Hz, CH); 139.0 (s, C); 139.3 (s, C); 140.2 (s, C); 141.5 (s, C) ppm. ^31^P-{^1^H} NMR (202 MHz, CDCl_3_): δ = 41.1 (s) ppm. HRMS (ESI+, *m/z*) [M + Li]^+^ calculated 577.0738 found 577.0742.

(**2**)Au(NTf_2_) was synthesized according to the previously reported procedure [19]. ^1^H NMR (500 MHz, CDCl_3_): δ = 3.46–3.57 (m, 1H); 3.79–3.93 (m, 1H); 4.99–5.01 (m, 1H); 7.49–7.76 (m, 14H); 7.80–7.87 (m, 2H) ppm. ^13^C NMR (125 MHz, CDCl_3_): δ = 35.4 (d, J = 6.1 Hz, CH_2_); 40.0 (d, J = 33.3 Hz, CH); 118.9 (q, J = 21.8 Hz, CF_3_); 120.0 (s, CH); 120.7 (s, C); 120.9 (d, J = 6.9 Hz; CH); 123.1 (s, CH); 124.2 (d, J = 3.6Hz, CH); 127.4 (d, J = 2.8 Hz, CH); 127.8 (s, CH); 129.1 (d, J = 11.7 Hz, CH); 129.8 (d, J = 11.6 Hz, CH); 131.1 (s, C); 132.0 (s, CH); 132.8 (s, CH); 133.2 (d, J = 12.4 Hz, CH); 134.5 (d, J = 13.1 Hz, CH); 138.3 (d, J = 3.6 Hz, C); 139.3 (s, CH); 140.2 (s, CH) ppm. ^31^P-{^1^H} NMR (202 MHz, CDCl_3_): δ = 47 (s) ppm. ^19^F NMR (470 MHz, CDCl_3_): δ = −79 (s) ppm.

### 4.6. Synthesis of Silver Complex **2_Ag_**

Phosphine **2** (100 mg; 0.30 mmol) was added to a suspension of silver chloride (85 mg; 0.60 mmol) in anhydrous dichloromethane. The mixture was placed in the dark and stirred vigorously at 50 °C under an argon atmosphere for 48 h. The reaction mixture was diluted with 2 mL of anhydrous dichloromethane and filtered. The supernatant was collected, the solvent was evaporated, and the solid was dried under reduced pressure. Complex **2_Ag_** was obtained after recrystallisation from dichloromethane/*n*-hexane (62 mg; 44%).

^1^H NMR (500 MHz, (CD_3_)_2_SO): δ = 3.27–3.38 (m, 2H); 3.66–3.77 (m, 2H); 5.23–5.30 (m, 2H); 7.24–7.30 (m, 4H); 7.43–7.67 (m, 20H); 7.78–7.93 (m, 8H) ppm. ^13^C NMR (125 MHz, (CD_3_)_2_SO): δ = 36.2 (d, J = 10.7 Hz, CH_2_); 39.4 (d, J = 18.1 Hz, CH); 120.1 (s, CH); 123.3 (s, CH); 124.2 (s, CH); 127.8 (s, CH); 129.3 (s, CH); 130.5 (t, J = 9.0 Hz, CH); 131.1 (s, CH); 132.1 (s, C); 132.2 (s, CH); 134.0 (d, J = 15.8 Hz, CH); 135.4 (d, J = 16.5 Hz, CH); 139.3 (s, C); 139.2 (s, C); 143.2 (s, C); 144.2 (s, C) ppm. ^31^P-{^1^H} NMR (202 MHz, (CD_3_)_2_SO): δ = 18 (large singlet) ppm. MS (ESI+, *m/z*) [C_24_H_19_AgP]^+^ 445.07. Elem. anal. Calcd: C, 59.84; H, 3.98. Found: C, 59.99; H, 4.06.

### 4.7. Synthesis of Copper Complex **2_Cu_**

Phosphine **2** (150 mg; 0.44 mmol) was added to a suspension of CuBr(SMe_2_) (91 mg; 0.44 mmol) in anhydrous dichloromethane. The mixture was stirred vigorously at R.T. under an argon atmosphere for 0.5 h. The solvent was evaporated under vacuum and the solid was washed with *n*-hexane. Complex **2_Cu_** was obtained after recrystallisation from dichloromethane/*n*-hexane (90 mg; 42%).

^1^H NMR (500 MHz, (CD_3_)_2_SO): δ = 3.42–3.52 (m, 2H); 3.59–3.70 (m, 2H); 5.07–5.15 (m, 2H); 7.22–7.27 (m, 4H); 7.41–7.56 (m, 16H); 7.61 (d, J = 8.1 Hz, 4H); 7.82–7.95 (m, 8H) ppm. ^13^C NMR (125 MHz, (CD_3_)_2_SO): δ = 36.3 (d, J = 11.0 Hz, CH_2_); 39.1 (d, J = 19.4 Hz; CH); 120 (CH); 123 (CH); 124 (CH); 128.0 (CH); 128.5 (CH); 129.4 (CH); 129.9 (CH); 131 (CH); 131.5 (CH); 132.2 (C); 134.7 (d, J = 14.3 Hz, CH); 135.2 (d, J = 15.3 Hz, CH); 140.1 (C); 140.7 (C); 144.5 (C); 144.0 (C) ppm. ^31^P-{^1^H} NMR (202 MHz, (CD_3_)_2_SO): δ = 1 (large singlet) ppm. MS (ESI+, *m/z*) [C_24_H_19_BrCuLiP]^+^ 487.00.

### 4.8. Synthesis of Palladium Complexes **2_Pd1_** and **2_Pd2_**

The complexes were obtained with a reaction of [Pd(allyl/indenyl)Cl]_2_ with 2 equivalents of phosphine **2** in dry diethylether under nitrogen overnight. The resulting solid was isolated using filtration and was washed with cyclohexane. A recrystallisation was then carried out from slow diffusion of *n*-pentane in a concentrated solution of the complex in CH_2_Cl_2_.

**2_Pd1_**. (169 mg, 84%) starting from **2** (130 mg, 0.384 mmol), [Pd(allyl)Cl]_2_ (91 mg, 0.248 mmol), and 10 mL of diethylether. ^1^H NMR (500 MHz, CDCl_3_): δ = 2.23 (dd, J = 12.1 Hz, J = 43.2 Hz, 1H), 2.98 (dd, J = 6.8 Hz, J = 18.7 Hz, 1H), 3.43–3.73 (m, 2H), 3.81–3.95 (m, 1H), 4.60–4.74 (m, 1H), 5.05 (m, 1H, CH_central_ major isomer), 5.25 (m, 1H, major isomer), 5.29 (m, 1H, minor isomer), 5.38 (m, 1H, CH_central_ minor isomer), 6.96–7.64 (m, 16H) ppm. ^13^C NMR (125 MHz, CDCl_3_): δ = 35.7 (d, J = 16.9 Hz, CH_2_, minor isomer); 35.8 (d, J = 18.6 Hz, CH_2_, major isomer); 40.2 (d, J = 19.4 Hz, CH, minor isomer); 40.5 (d, J = 19.0 Hz, CH, major isomer); 57.1 (s, CH_2_, major isomer, C_allyl_); 57.9 (s, CH_2_, minor isomer, C_allyl_); 79.5 (d, J = 25.5 Hz, CH_2_, C_allyl_, minor isomer); 79.8 (d, J = 25.4 Hz, CH_2_, C_allyl_, major isomer); 117.1 (s, C_allyl_, minor isomer); 117.2 (s, C_allyl_, major isomer); 119.4–143 (C_arom_). ^31^P-{^1^H}-NMR (202 MHz, CDCl_3_): δ 36.1 (minor isomer), 37.0 (major isomer) ppm. Elem. anal. Calcd: C, 62.21; H, 4.64. Found: C, 61.86; H, 4.46.

**2_Pd2_**. (118 mg, 68%) starting from **2** (98 mg, 0.289 mmol), [Pd(indenyl)Cl]_2_ (102 mg, 0.200 mmol) and 10 mL of diethylether. ^1^H NMR (500 MHz, CDCl_3_): δ = 3.30–3.50 (m, 1H), 3.62–3.80 (m, 1H), 5.05–5.19 (m, 1H), 6.02–6.07 (m, 1H), 6.42–6.54 (m, 2H), 6.68–7.61 (m, 20H) ppm. ^13^C NMR (125 MHz, CDCl_3_): δ = 35.6 (s, CH_2_); 40.9 (d, J = 21.6 Hz, CH); 75.2 (d, J = 3.7 Hz, CH_ind_); 96.6 (d, J = 23.1 Hz, CH_ind_); 111.1 (d, J = 6.2 Hz, CH_ind_); 117.1–135.5 (CH_arom_); 136.5 (d, J = 4.8 Hz, C_q,Ind_); 139.9 (d, J = 4.6 Hz, C_q,Ind_); 142.6 (s, C_q,arom_). ^31^P-{^1^H}-NMR (202 MHz, CDCl_3_): δ = 40.6 ppm. Elem. anal. Calcd: C, 66.57; H, 4.40. Found: C, 66.29; H, 4.51.

## 5. Conclusions

We have shown that the direct reaction of diphenylphosphine with a cyclic internal alkene can generate tertiary phosphines of interest for coordination chemistry and homogeneous catalysis. In particular, the reaction of diphenylphosphine with acenaphthylene gives ligand **2**, which has an interesting positioning in regard to 1,2-dihydroacenaphthylene relative to the metal, as deduced from X-ray diffraction studies of the synthesized metal complexes. In these complexes, dihydroacenaphthylene is close to the first coordination sphere of the metal. In order to improve the catalytic performance of these systems, it would now be interesting to introduce much more bulky substituents on phosphorus (i.e., *^t^*Bu or Adamantyl vs. Ph).

## Figures and Tables

**Table 1:** Suzuki–Miyaura cross-coupling reaction and Buchwald-Hartwig reaction with ligand **2**.

Entry *	Substrate 1	Substrate 2	Product	Yield (%)
1	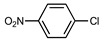	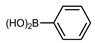	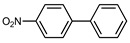	96
2	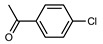		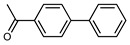	99
3	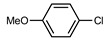		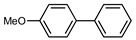	98
4	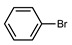	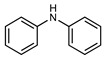	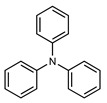	30
5	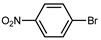		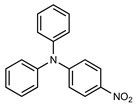	44

* See Supplementary Materials for experimental details.

## Data Availability

Data are contained within the article and Supplementary Materials.

## Supplementary Materials

The following supporting information can be downloaded at: https://www.mdpi.com/article/10.3390/molecules29163946/s1, Figure S1: Single-crystal X-ray molecular structure of phosphine oxide of ligand **1**; Figure S2: Single-crystal X-ray molecular structure of ligand **2**. General procedures for the catalytic tests.
