# Supplementary material for: Synthesis and Characterization of Transition Metal Complexes Supported by Phosphorus Ligands Obtained Using Hydrophosphination of Cyclic Internal Alkenes
Source: Molecules. 2024 Aug 21;29(16):3946. doi: 10.3390/molecules29163946 (PMC11356854; doi:10.3390/molecules29163946)

**Supplementary information of**

# Synthesis and characterization of transition metal complexes supported by phosphorus ligands obtained by hydrophosphination of cyclic internal alkenes.

**Victoria Mechrouk [1], Damien Bissessar[1], Julien Egly[1], Jordan Parmentier [1] and Stéphane Bellemin-Laponnaz [1,*]**

[1] Institut de Physique et Chimie des Matériaux de Strasbourg, Université de Strasbourg-CNRS UMR7504, 23 rue du Loess, BP 43, CEDEX 2, 67034 Strasbourg, France

* Correspondence: bellemin@unistra.fr

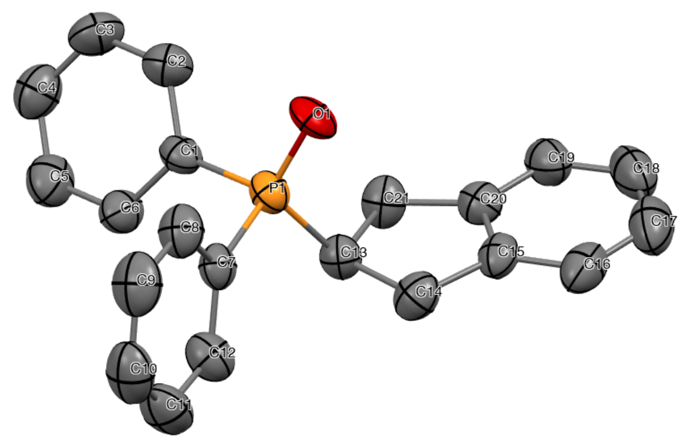


**Figure S1.** Single-crystal X-ray molecular structure of ligand **1** (oxidized form). Hydrogen atoms are omitted for clarity. Space group: orthorhombic P b c a. Cell length a 9.6619(10), b 10.7232(10), c 32.600(3). Crystal obtained from slow evaporation of a dichloromethane solution of **1** in air. CCDC deposit number **2373137**.

Selected bond distances (Å) and angles (°): P(1)-O(1), 1.481(3); P(1)-C(13), 1.795(4); P(1)-C(1), 1.790(4); P(1)-C(7), 1.804(4); O(1)-P(1)-C(1), 111.07(18); O(1)-P(1)-C(13), 114.59(18); C(1)-P(1)-C(13), 107.23(19); O(1)-P(1)-C(7), 112.17(18); C(1)-P(1)-C(7), 104.10(17); C(13)-P(1)-C(7), 107.02(19).

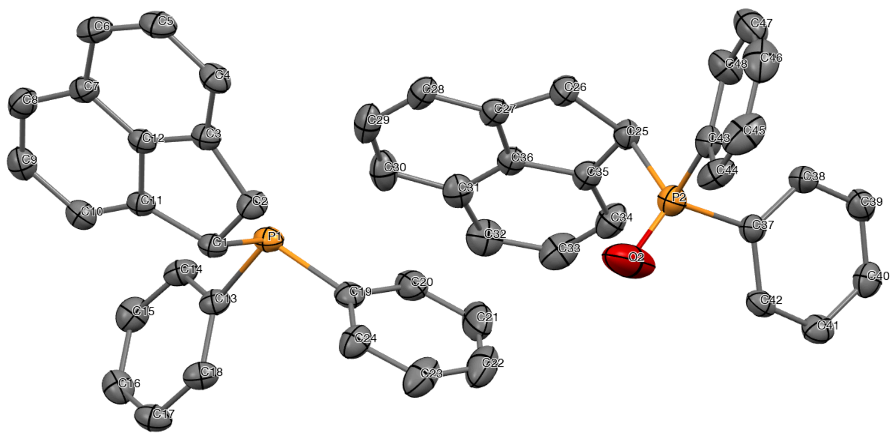


**Figure S2.** Single-crystal X-ray molecular structure of ligand **2**. The unit cell contains ligand **2** and its oxidized form. Space group: triclinic P -1. Cell length a 10.8105(5), b 11.5236(5), c 16.7521(7); alpha 88.7100(10), beta 75.5630(10), gamma 62.7720(14). Crystal obtained from slow evaporation of a dichloromethane solution of **2** in air. CCDC deposit number **2373142**.

Selected bond distances (Å) and angles (°): C(1)-P(1), 1.863(2); C(13)-P(1), 1.835(2); C(19)-P(1), 1.835(2); C(25)-P(2), 1.851(2); C(37)-P(2), 1.828(2); C(43)-P(2), 1.827(2); O(2)-P(2), 1.431(5); C(13)-P(1), C(19), 102.62(9); C(13)-P(1)-C(1), 99.18(9); C(19)-P(1)-C(1), 102.26(9); O(2)-P(2)-C(43), 114.0(2); O(2)-P(2)-C(37), 114.0(3); C(43)-P(2)-C(37), 104.88(10); O(2)-P(2)-C(25), 117.2(2); C(43)-P(2)-C(25), 103.24(10); C(37)-P(2)-C(25), 101.98(10).

**General procedure for the Suzuki-Miyaura cross-coupling of aryl halides**

Aryl boronic acid (0.55 mmol), $K_2CO_3$ (2.2 equiv.), 1 mol% of **2**, $[Pd(allyl)Cl]_2$ (0.5 mol%) and a stirring bar were added into a 4 mL vial in the dry box. Outside the glove box, aryl halide (0.5 mmol), degassed THF (1.6 mL) and water (0.4 mL) were added and the reaction was stirred at 80 °C for 12 hours. Afterwards, the reaction mixture was diluted with dichloromethane, washed with water and the organic layers were dried with anhydrous MgSO4. The product was isolated by chromatography on silica gel (EtOAc/cyclohexane).

**General procedure for the Buchwald-Hartwig coupling reaction**

In a glovebox, diphenylamine (100 mg, 0.59 mmol), 4-bromoanisole (122 mg, 0.65 mmol), phosphine **2** (4.0 mg; 0.012 mmol), $Pd(OAc)_2$ (1.3 mg, 0.006 mmol) and NaOtBu (68 mg, 0.71 mmol) are mixed in a screw-top vial. The resulting mixture is heated at 110°C for 18 h. After cooling, the reaction mixture is diluted in a 1:1 dichloromethane-water mixture and the organic phase recovered. The organic phases are combined and concentrated under reduced pressure. The product was isolated by chromatography on silica gel (EtOAc/cyclohexane).

**General procedure for the gold-catalyzed reactions: hydroamination and hydration**

(**2**)$AuNTs_2$ was prepared according to: Mézailles, N.; Ricard, L.; Gagosz, F. Phosphine Gold(I) Bis-(Trifluoromethanesulfonyl)Imidate Complexes as New Highly Efficient and Air-Stable Catalysts for the Cycloisomerization of Enynes. *Org. Lett.* **2005**, *7*, 4133–4136.

The procedure was followed from: a) Mizushima, E.; Hayashi, T.; Tanaka, M. Au(I)-Catalyzed Highly Efficient Intermolecular Hydroamination of Alkynes. *Org. Lett.* **2003**, *5*, 18, 3349–3352. b) Mizushima, E.; Sato, K.; Hayashi, T.; Tanaka, M. Highly Efficient $Au^{I}$-Catalyzed Hydration of Alkynes. Angew. Chem. Int. Ed. **2002**, 41, 4563–4565.

Ligand **2**

$^1$H NMR (300 MHz, $CD_2Cl_2$)

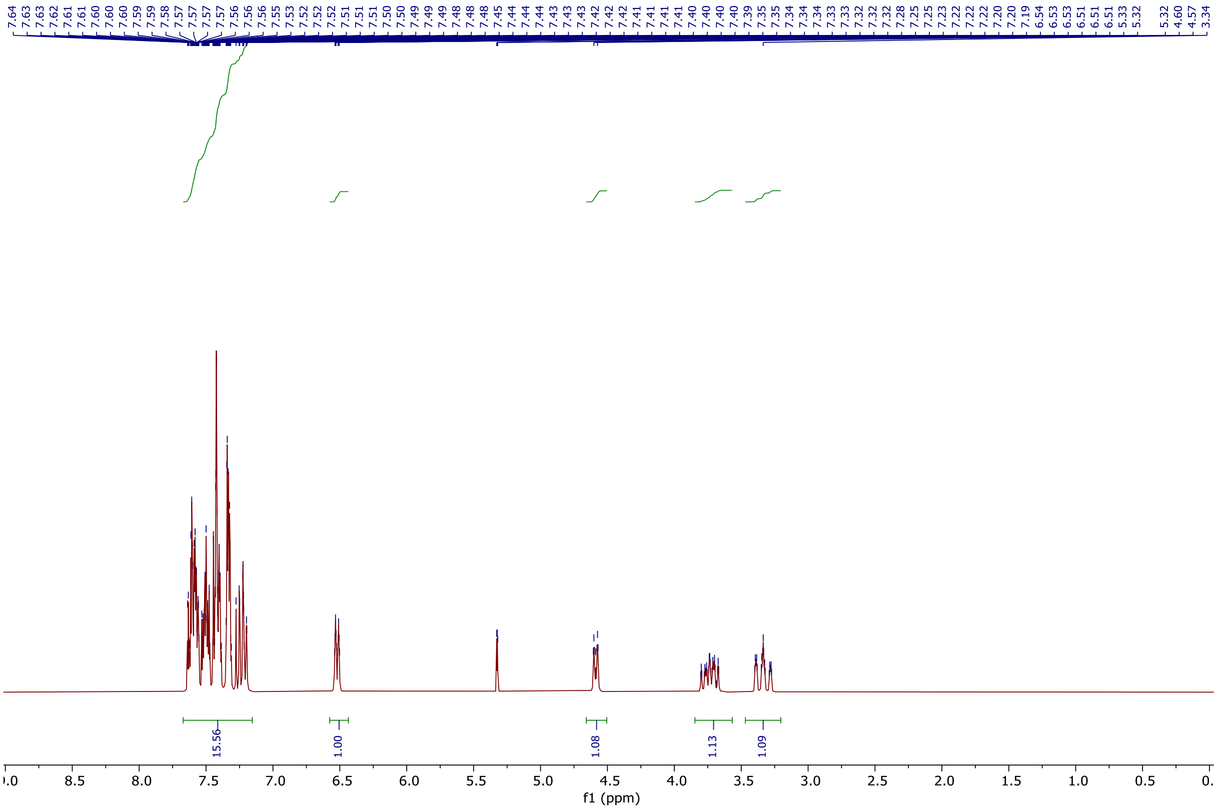

$^{13}C$ NMR (75 MHz, $CDCl_3$)

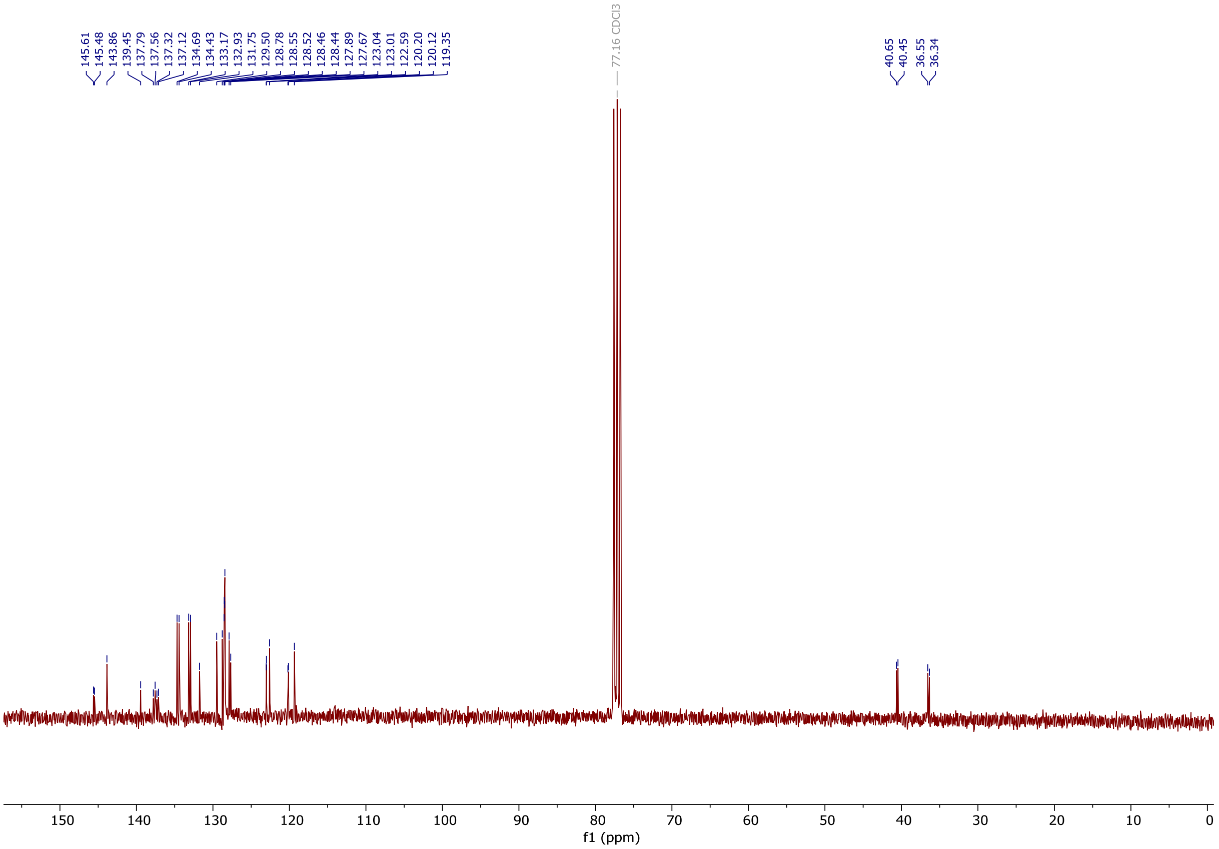


$^{31}P$ NMR (121 MHz, $CD_2Cl_2$)

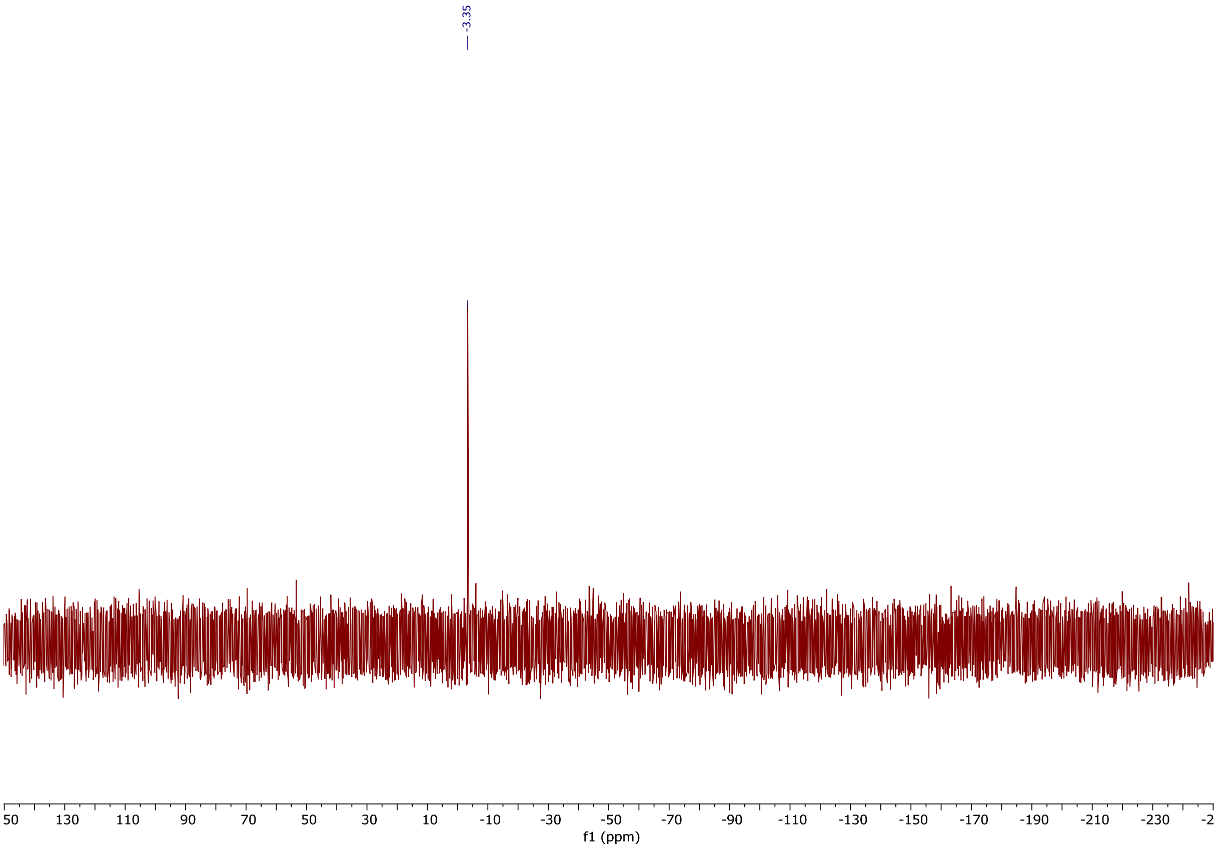

Complex $\mathbf{2}_{\mathbf{Pd1}}$

$^{1}$H NMR (500 MHz, $CDCl_3$)

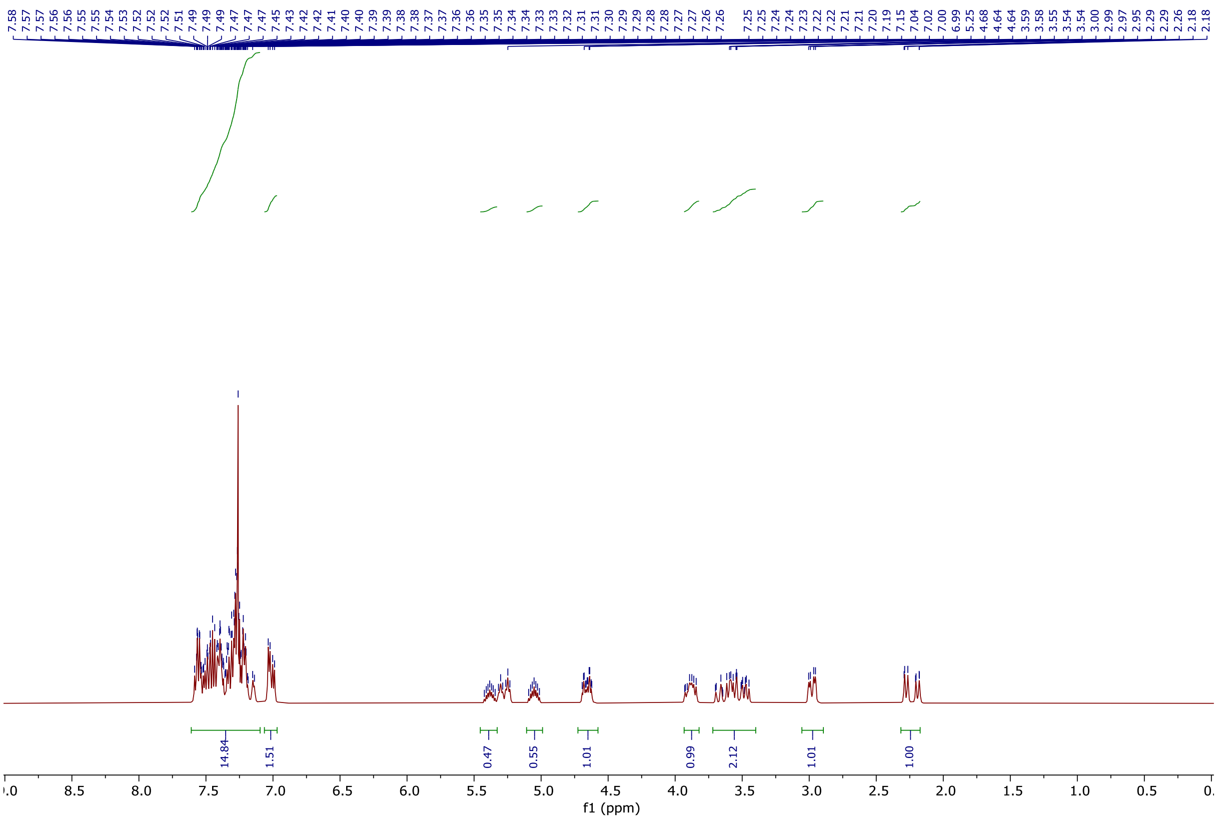

$^{13}C$ NMR (125 MHz, $CDCl_3$)

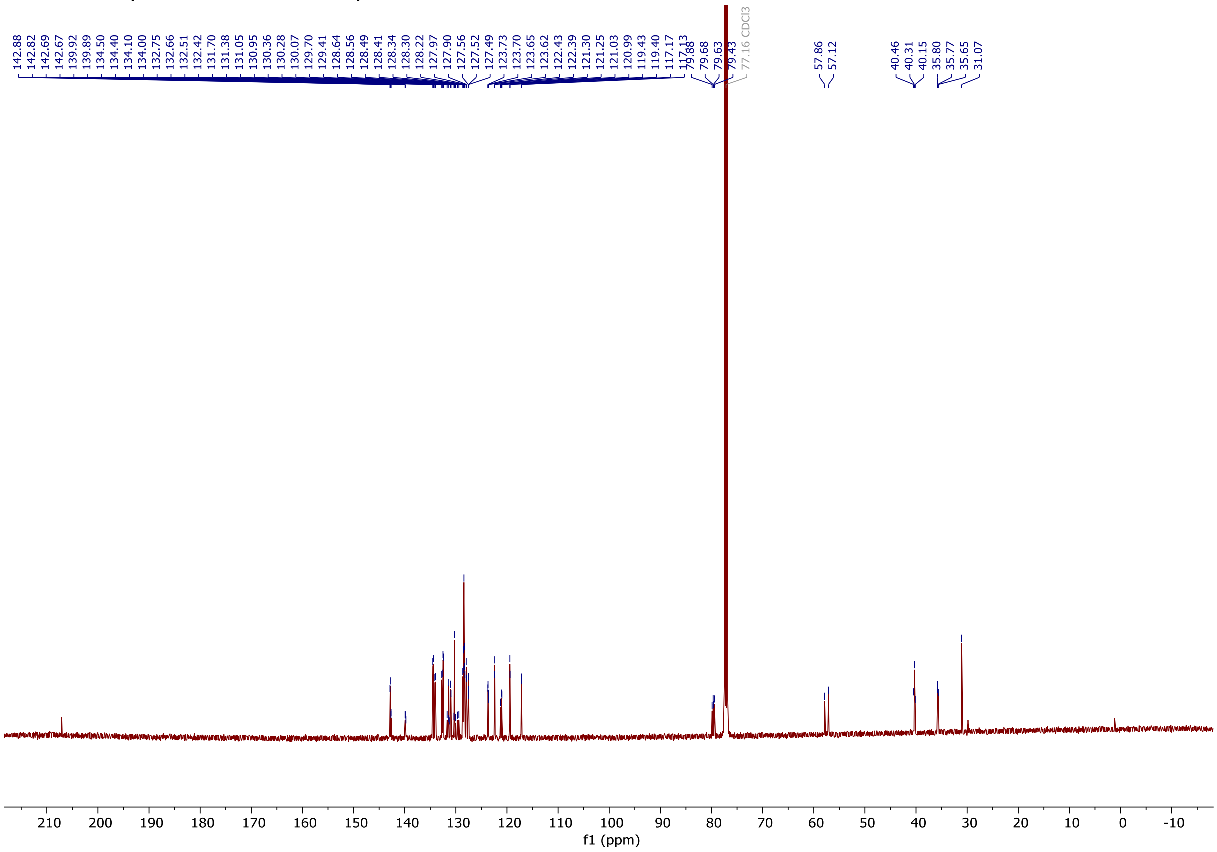


$^{31}P$ NMR (202 MHz, $CDCl_3$)

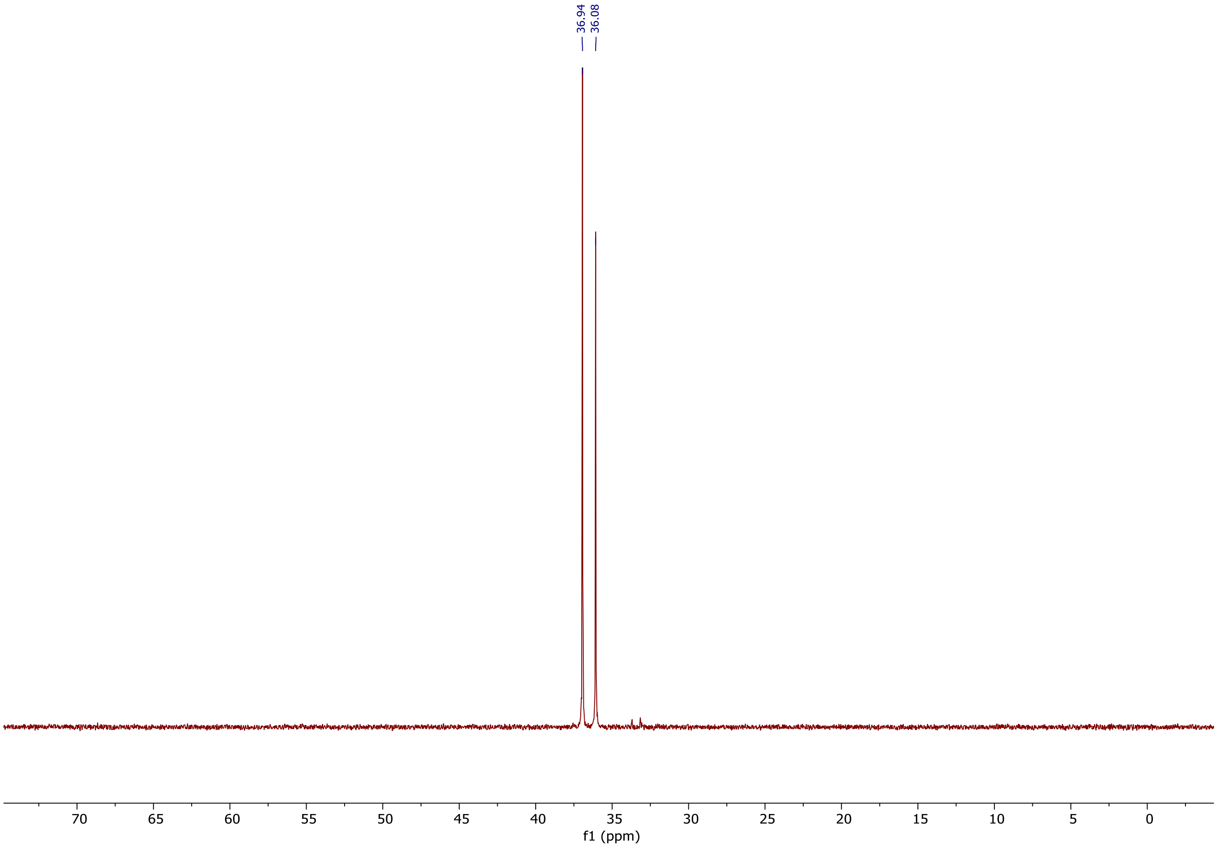

Complex $\mathbf{2_{Pd2}}$

$^1$H NMR (500 MHz, $CDCl_3$)

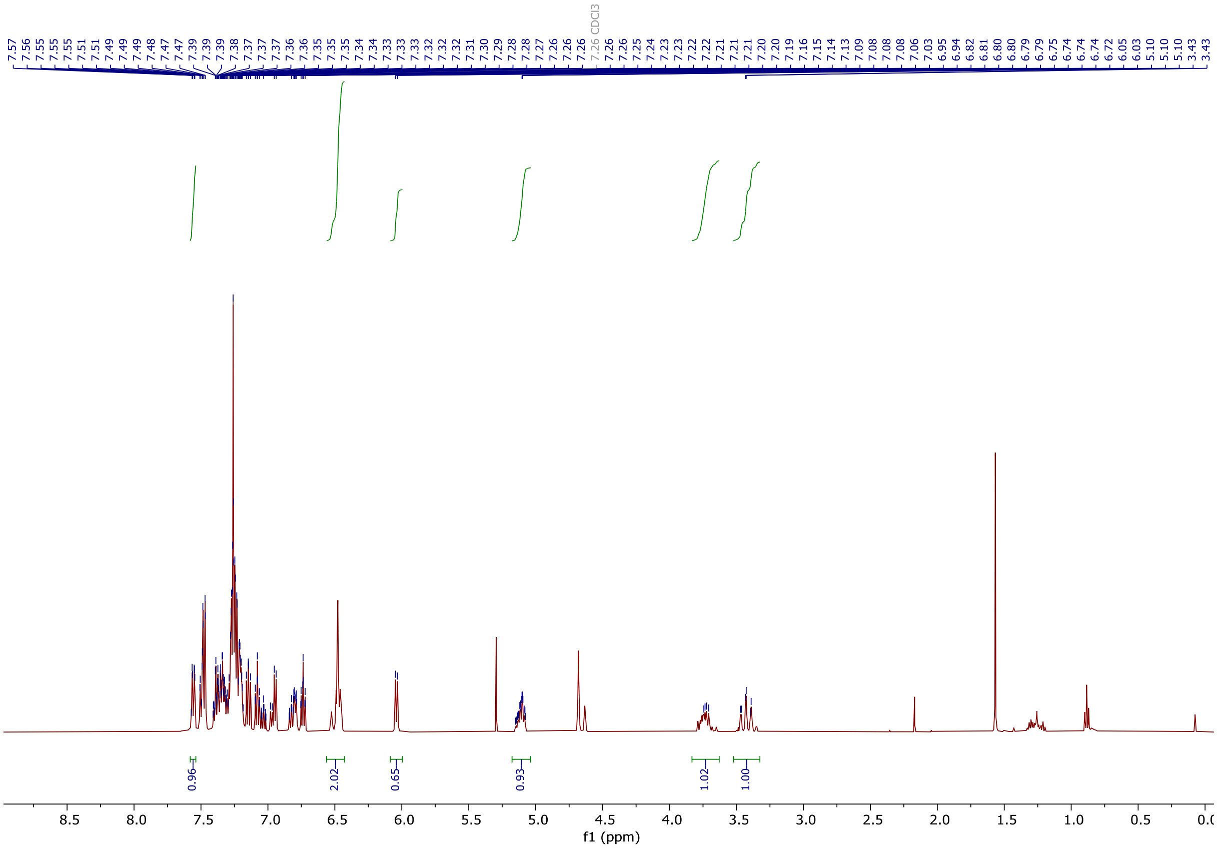

$^{13}$C NMR (125 MHz, $CDCl_3$)

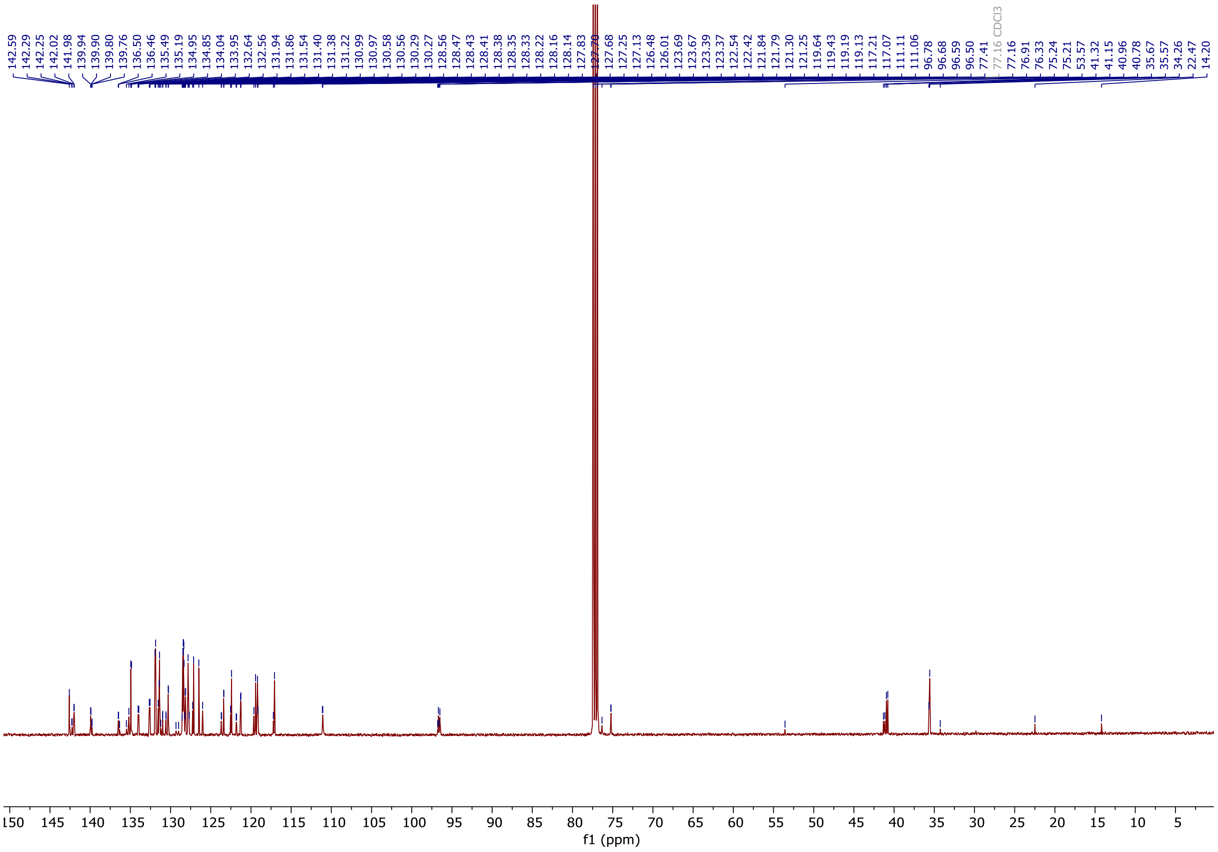


$^{31}$P NMR (202 MHz, $CDCl_3$)

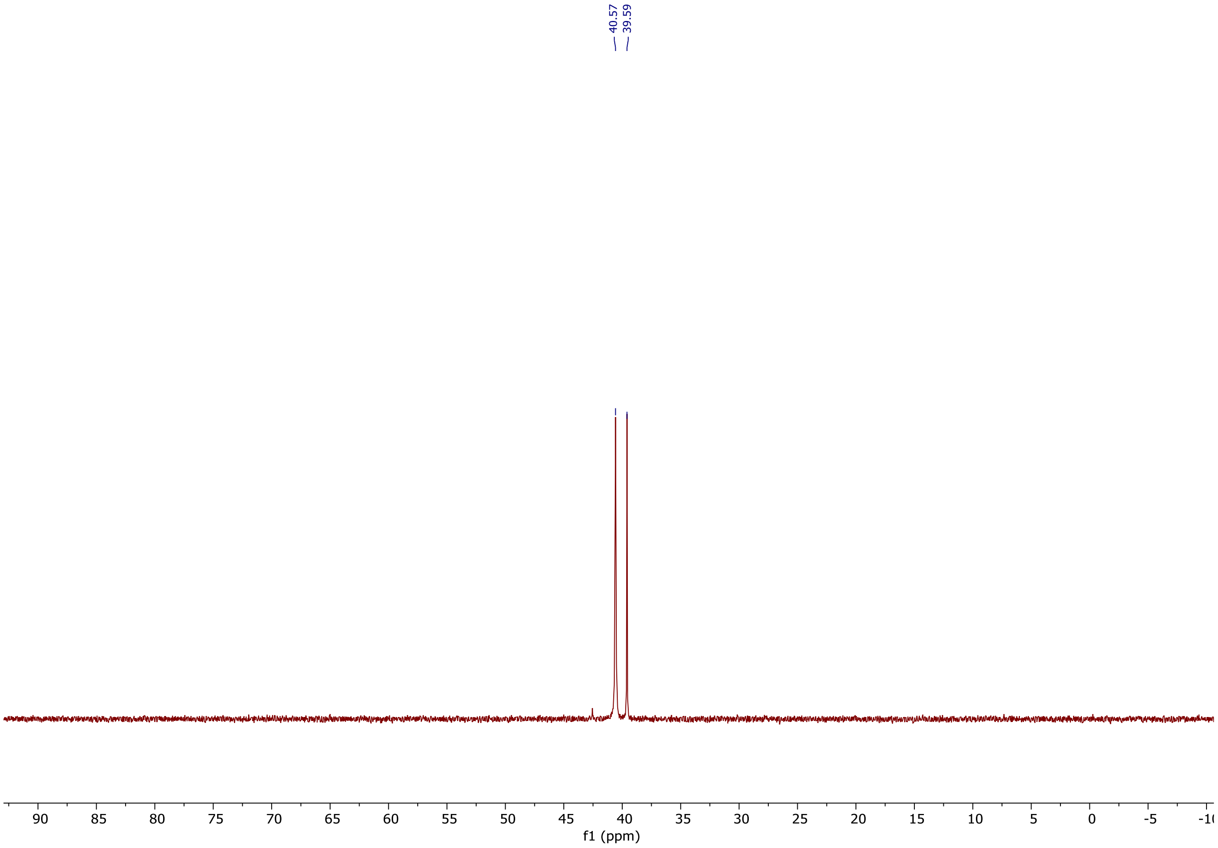

Supplement: Supplementary file 1 [file molecules-29-03946-s001.zip › molecules-3149240-supplementary.pdf]
